# Design Strategies for Forward Osmosis Membrane Substrates with Low Structural Parameters—A Review

**DOI:** 10.3390/membranes13010073

**Published:** 2023-01-07

**Authors:** KmProttoy Shariar Piash, Oishi Sanyal

**Affiliations:** Department of Chemical and Biomedical Engineering, West Virginia University, Morgantown, WV 26506, USA

**Keywords:** forward osmosis membrane, transport parameters, structural parameter, support layer modification, internal concentration polarization

## Abstract

This article reviews the many innovative strategies that have been developed to specifically design the support layers of forward osmosis (FO) membranes. Forward osmosis (FO) is one of the most viable separation technologies to treat hypersaline wastewater, but its successful deployment requires the development of new membrane materials beyond existing desalination membranes. Specifically, designing the FO membrane support layers requires new engineering techniques to minimize the internal concentration polarization (ICP) effects encountered in cases of FO. In this paper, we have reviewed several such techniques developed by different research groups and summarized the membrane transport properties corresponding to each approach. An important transport parameter that helps to compare the various approaches is the so-called structural parameter (S-value); a low S-value typically corresponds to low ICP. Strategies such as electrospinning, solvent casting, and hollow fiber spinning, have been developed by prior researchers—all of them aimed at lowering this S-value. We also reviewed the quantitative methods described in the literature, to evaluate the separation properties of FO membranes. Lastly, we have highlighted some key research gaps, and provided suggestions for potential strategies that researchers could adopt to enable easy comparison of FO membranes.

## 1. Introduction

**Figure 1:**
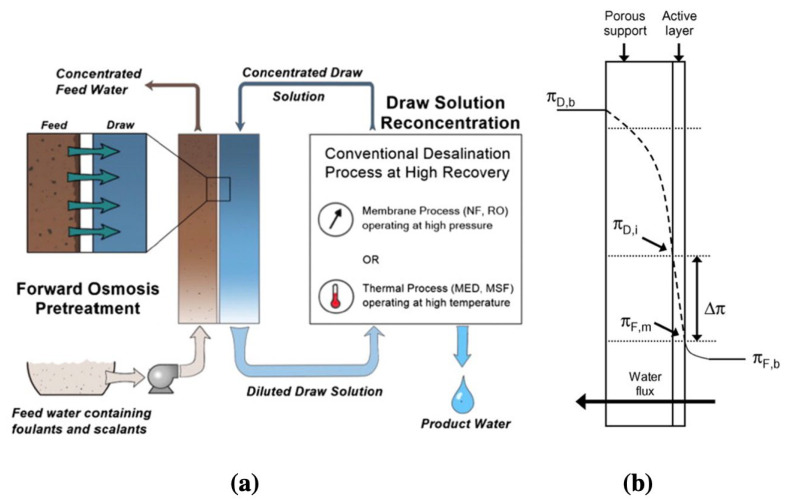
(**a**) Schematic representation of a forward osmosis process (reproduced from Shaffer et al. [15] with permission from Elsevier); and (**b**) Osmotic pressure gradient in an asymmetric forward osmosis membrane where the active layer facing feed solution is incorporating both ICP and ECP (reproduced from McCutcheon et al. [16] with permission from Elsevier). In Figure 1b, $\pi_{D,b\;}$ and $\pi_{F,b}\;$ are the bulk osmotic pressures of the draw solution and feed solution, respectively, while $\pi_{D,i\;}$ and $\pi_{F,m}$ are the effective osmotic pressures.

Extraction of natural gas from unconventional shale reservoirs via hydraulic fracturing has revitalized the energy industry and is often referred to as a “*game-changer*” for the US energy landscape [1]. The drilling and fracturing activities, however, lead to large quantities of hypersaline-produced water, ranging from ~25-gallon water/MMcf (million cubic feet) of gas produced to 1000-gallon/MMcf, depending on the shale source and well lifetime [2,3]. Sustainable wastewater management is, therefore, critical to address the detrimental environmental effects of hypersaline water discharge, as well as for the long-term economic viability of this technology. In the case of seawater (TDS ~35,000 mg/L; equivalent to 70,000 mg/L with 50% recovery) desalination and brackish water reclamation, reverse osmosis (RO) has been essentially established as the dominant separation technique. The level of total dissolved solids (TDS) in produced water, however, range from ~1000 to 463,000 ppm [4,5,6,7,8]. The hydraulic pressure needed to overcome the high osmotic pressure related to high-salinity feed streams, exceeds the practical working limits of polyamide thin-film composite (PA-TFC) membranes [9], which are state-of-the-art for RO membranes. Forward osmosis (FO), which is an osmotically driven separation process, is a viable alternative to reverse osmosis (RO) to treat high-salinity water sources [10,11,12,13,14]. FO is a chemical-potential driven process where semipermeable membranes are used to separate water from dissolved solutes. One side of the semipermeable membrane is in contact with the feed solution (the high salinity produced water treatment in this case), while the other side faces a highly concentrated “*draw solution*”, with lower water potential than the feed solution (Figure 1a). Unlike RO, where hydraulic pressure is required to overcome the osmotic pressure differential between the feed and the permeate, FO is solely driven by the chemical-potential difference between the two solutions.

The draw solution, which is diluted over time with the passage of pure water from the feed to the draw side, is eventually regenerated to produce pure water as the product. Ultimately, successful deployment of the FO process for water desalination production relies on both improved membrane design as well as the selection of appropriate draw solutions. In this article, we will primarily focus on the membrane material aspect of FO processes, while the readers are directed towards other references that review the various draw solution selections [17,18,19,20,21].

A typical forward osmosis membrane type is a thin-film composite (TFC) consisting of a porous support layer (substrate) and an active layer (selective layer). Depending on whether the active layer faces the feed or the draw solution, osmotic processes, such as FO and pressure-retarded osmosis (PRO), could be carried out under either of the two operational modes—AL-FS mode, where the active layer faces the feed solution, and AL-DS mode, where the active layer faces the draw solution [16,22]. AL-FS is more common for FO mode, since it lowers the membrane fouling propensity [23,24]. In this article, we primarily review the results of FO membranes and processes carried out under AL-FS mode. FO membrane fouling and its mitigation strategies are also outside the scope of this paper and have been reviewed elsewhere [24,25,26,27,28].

Unlike traditional pressure-driven membrane processes, the performance of FO membranes largely depend on the porous support layer’s structure [29,30,31]. This is due to the unique internal concentration polarization (ICP) effect encountered in the case of FO processes (Figure 1b). The usual concentration polarization phenomenon that occurs in case of all membrane processes, resulting in a build-up of rejected solute near the membrane selective layer, occurs in the case of FO processes as well. However, an additional *internal concentration polarization* effect is observed in the case of FO membranes, due to dilution of the draw solution within the support layer. The simultaneous occurrence of both these concentration polarization effects naturally leads to very low fluxes for FO membranes. To distinguish between these two polarization effects, the usual concentration polarization is referred to as external concentration polarization (ECP), while the concentration dilution within the support layer is referred to as *internal concentration polarization* (ICP). As Figure 1b shows, while the ideal osmotic pressure differential is ($\pi_{D,b\;}–\;\pi_{F,b\;}),$ the effective osmotic pressure ends up being ($\pi_{D,i\;}–\;\pi_{F,m\;})$, because of the concentration polarization effects, essentially reducing the driving force significantly. For a detailed mathematical framework of these polarization effects, the readers are directed to the article by McCutcheon et al. [16].

While ECP can be countered by controlling the hydrodynamic conditions, such as cross flow rate, stirring rate, etc., ICP is difficult to mitigate since it occurs due to an unstirred boundary layer formation within the support layer [16]. The ICP effect reduces the overall driving force for the membrane, which could be partially countered by the back-diffusion of the draw solutes. The rate of this back-diffusion, however, is influenced by the properties of the membrane support layer, which typically faces the draw solution in the case of FO processes. Unlike pressure-driven membrane processes, the membrane support layer design is critical for osmotic processes, and in fact, the support layers of conventional NF/RO membranes are not suitable for FO processes. Novel support layer designs are, therefore, critical to ensure high water permeabilities across FO membranes and this forms the focus of this review article.

### 1.1. Structural Parameter (S) and Its Role in Reducing ICP Effects in FO Membranes

The dilutive ICP effect is described by the following equation [16]:

(1) $$J_{W}=A\;[\pi_{D,b}\exp\left(-J_{W}K\right)-\pi_{F,b}\exp\left(\frac{J_{W}}{K}\right)]$$

In the equation above, $J_{w}$ refers to the permeate water flux across the membrane, while K here refers to the solute resistivity for diffusion. K is related to both the membrane morphological properties as well as the solute diffusion coefficient, via the relation, K=τ t/ε D, where τ = support layer tortuosity, t = support layer thickness, ε= support layer porosity, and D = solute diffusion coefficient.

The term (τ t/ε), collectively, is the *structural parameter* (S-value) of the FO membrane. Since the solute diffusion coefficient (D) is fixed for a certain membrane material under specific conditions, the solute mass transfer ultimately depends on the S-value, viz., the thickness, porosity, and tortuosity of the support layer. The S-value has generally been accepted as an easy metric to compare different FO membranes, even when tested under varied experimental conditions. A low S-value relates to reduced ICP effects [32,33,34] and, as described in the later sections, research in this area is primarily focused on minimizing the support S-values via the reduction in thickness, tortuosity and increase in porosity. Reducing the thickness beyond a certain level could compromise the mechanical integrity of the membrane. Fortunately, a computational fluid dynamics study on hollow fiber membrane modules by Ren et al. [35] demonstrated that ultrathin support layers may not be necessary for high performance FO membranes, as reducing the support layer thickness by 50% (from 200 to 100 µm) only resulted in 7% water flux enhancement. The contact angle of the membrane surface, which quantifies wettability, is not a direct parameter included in the relation for S-value, however, McCutcheon et al. [36] highlighted the critical role of support hydrophilicity in limiting the ICP effects. Improved hydrophilicity of membrane support enhances pore wetting, which in return improves water flux by decreasing support layer resistance and ICP effects. In other words, higher membrane hydrophilicity essentially leads to higher effective porosity and pore connectivity, which brings the S-value down even when no apparent changes in the porous structure are evident. This fact will be evident from the discussions related to novel fabrication strategies for S-value reduction in later sections of this paper.

Some of the most common fabrication strategies used by researchers to design low- S-value supports include electrospinning, phase inversion/solvent casting, and hollow fiber spinning. In this article, we have reviewed and compared these support layer fabrication techniques based on their water and salt permeabilities, fluxes, and support layer S-values. Difficulties in comparing permeability values, however, occur due to widely different testing conditions used by different research groups. Since the S-value is generally assumed to be independent of the testing conditions, it allows a meaningful comparison for the support layer fabrication strategies. Although a low S-value helps to reduce the ICP effects, the final flux/permeability value of the FO membrane also depends on the draw solution composition and selective layer properties among other factors. To date, there are several articles that have reviewed different aspects of FO, such as methods and process development [37,38,39,40,41,42,43,44,45,46], applications [47,48,49,50,51,52,53,54], fouling behaviors [24,26,55,56,57,58,59], draw solution characteristics [17,18,19,20,21,60,61], and energy considerations [15,62,63,64,65,66,67]. There are, however, few studies that provide a focused overview of the different support layer design strategies [25,68] along with the diverse transport property evaluation methodologies [69] that are discussed in the literature. This review will critically analyze the various support layer fabrication techniques that are consequential in determining the performance of FO membranes, primarily via reduced S-values. The methods used to determine the intrinsic transport properties (i.e., water and salt permeability and S-value) of the membrane also vary to some extent between research teams. For the sake of brevity, we have reviewed the three most common experimental methods as well as two analytical methods for the evaluation of these properties.

## 2. Methods for Measuring the Transport Parameters of FO Membrane

The three parameters that fully describe the forward osmosis membrane’s performance are the water permeability (A), salt permeability (B), and the structural parameter (S). The following sub-sections summarize the experimental techniques used by different research groups to evaluate these parameters. Although some of these strategies are pure transport-based [70,71,72], a few analytical strategies, described by Kim et al. [73] and Manickam et al. [74], are also discussed in this section. A detailed review by Kim et al. [69], focusing exclusively on the FO S-value estimation, is available for anyone interested in the in-depth analyses of these strategies.

### 2.1. Transport-Based “Indirect” Techniques

#### 2.1.1. RO-FO-Based Method

(a) Pressurized RO-FO method

The pressurized RO-FO-based method is the most common technique to evaluate the transport parameters of FO membranes. In this method, water and salt permeabilities (A and B) are measured using an RO system, whereas the S-value is calculated by the FO mode. In this article, we designate this method as the “pressurized RO-FO” method to differentiate against the “low-pressure RO-FO” method described later, where much lower feed pressures were suggested. Cath et al. [71] proposed a standard methodology for testing high permeability osmotic-driven membrane processes to calculate the transport parameters using the RO-FO-based method. These standard conditions (Table 1) were used to create a “*round-robin*” experiment with 7 independent participating laboratories and the results were compared by Cath et al. It should be noted, though, that the recommended feed pressures were still lower than traditional brackish water (RO/seawater) RO processes (225–800 psi). It is typical to avoid higher pressures in the case of FO membranes since the support layers of these membranes are thin and lack robust mechanical properties, unlike RO membranes.

The following relations were used to evaluate the water and salt permeability coefficients (A and B) [34]:

(2) $$A\;=\frac{J_{w}}{P}$$

Here, $J_{w}$ is the water flux, P is the applied hydraulic pressure.

Salt permeability coefficient, B can be determined by this equation [75,76]:

(3) $$B=J_{w}\;(\frac{1-R}{R})\;exp\;\left(-\frac{J_{w}}{K}\right)\;$$

where R is the membrane rejection defined as R=1− $\frac{C_{p}}{C_{b}}$; $C_{P}$ and $C_{b}$ being the permeate and bulk concentrations, respectively.

**Figure 2:**
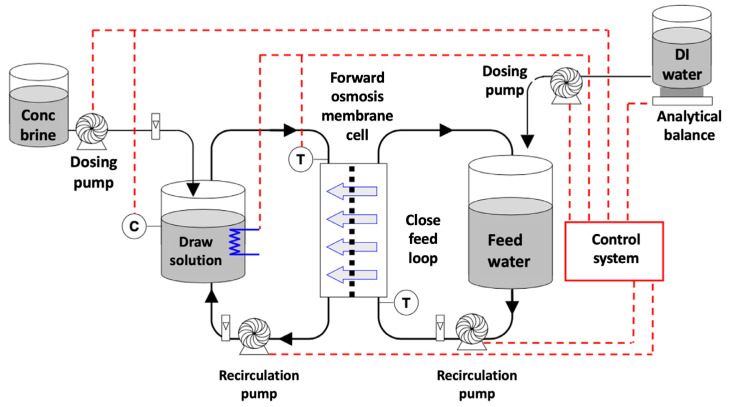
Bench-scale forward osmosis apparatus for calculating S-value (reproduced from Cath et al. [71] with permission from Elsevier).

The S-value is calculated under the FO mode where the conditions are noted in Table 1 and a standard experimental setup for FO processes used by Cath et al. [71], being shown below (Figure 2).

S-value was evaluated using the model below [77]:

(4) $$S=\frac{D}{J_{w}\;}\;\ln[\frac{B+A\;\pi_{D,b}}{B+J_{w}+A\;\pi_{F,m}\;}]\;$$

Here, D is the diffusivity of the draw solute. $\pi_{D,b}$ and $\pi_{F,m}$ are the osmotic pressures of the draw and feed solutions, respectively.

*“Round-robin*” experiments by seven different labs were conducted using two commercial membranes—a thin film composite (TFC) polyamide membrane and an asymmetric cellulose-based membrane [71]. The A values for both these membranes were consistent across (~9–~10 LMH/bar for TFC and ~0.8–~1.2 LMH/bar for cellulose) these labs. The B values of the TFC membrane showed more variability (~5$\times 10^{-7}$ to ~3 $\times 10^{-6}$ m/s) vs. the cellulose-based membrane (~1$\times 10^{-7}$ to ~3 $\times 10^{-7}$ m/s). The S-values for both TFC and cellulose-based membranes remained consistent (350–400 μm for TFC and 500–550 μm for cellulose-based) under FO mode. This highly rigorous effort by Cath et al., demonstrated that the “pressurized RO-FO method”, when performed under carefully controlled experimental conditions, produces very reproducible results.

(b) Ultra-low-pressure RO/FO-based method

FO supports are thin and highly porous; therefore, a high-pressure RO-method (described in Section 2.1.1a) to obtain A and B values may not be ideal for such membranes. Although a complete FO-based technique to evaluate the transport parameters would be ideal, as noted in Section 2.1.2, the technique becomes tedious, especially in lab-scale. As an alternative, Huang et al. [70] suggested evaluating parameter A with an ultra-low pressure (25 psi) RO method using Equation (2), and the parameters B and S using the standard FO process. The following two equations were solved simultaneously to evaluate B and S:

(5) $$J_{W}\;\;A\;\left\{\frac{\pi_{D,b}\;exp\;\left(-\frac{J_{w}\;S\;}{D}\;\right)-\pi_{F,b}\;exp\left(\frac{J_{w}}{K}\right)}{1+\frac{B}{J_{w}}\left[exp\left(\frac{J_{w}}{K}\right)-exp\;\left(-\frac{J_{w}\;S\;}{D}\;\right)\right]}\right\}$$

(6) $$J_{S}\;\;A\;\left\{\frac{C_{D,b}\;exp\;\left(-\frac{J_{w}\;S\;}{D}\;\right)-C_{F,b}\;exp\left(\frac{J_{w}}{K}\right)}{1+\frac{B}{J_{w}}\left[exp\left(\frac{J_{w}}{K}\right)-exp\left(-\frac{J_{w}\;S\;}{D}\;\right)\right]}\right\}\;$$

Here, $C_{D,b}$ and $C_{F,b}$ are the concentrations of the draw and feed solutions.

FO membranes are always operated at low osmotic pressures, thus a high hydraulic pressure for determining A and B would not be ideal and, in some cases, could lead to erroneous results. This ultra-low-pressure RO/FO method is preferred in that case. However, prior to wide-spread adoption of this method, it is recommended to compare and analyze the values obtained from this method vs. the pressurized RO-FO method.

#### 2.1.2. FO-Based Methods for Determining Transport Properties of FO Membranes

Tiraferri et al. [72] proposed a method to calculate all transport parameters (A, B, and S) using FO, instead of combining RO with FO. A unique experimental design was used in this case—solute-free DI water was used as the feed solution and a NaCl-based draw solution was used. The overall experiment was divided into 4 stages; where the draw solution concentration was increased progressively, and the steady-state flux values (water and solute) were recorded at every stage. Equations (5) and (6), described in Section 2.1.1b, were used to calculate the steady-state water and reverse solute fluxes for every stage.

For each of the 4 stages, two sets of values for $J_{W}$ and $J_{S}$ were obtained, in total, having a set of eight equations with three unknown parameters (A, B, and S). A rigorous least-square non-linear regression analysis was then performed to determine the parameters [72]. The results obtained from this technique were directly compared with the more prevalent “combined RO-FO” method and significant discrepancies were found—e.g., a ~46% average deviation was found in the membrane B values. The cause of this deviation was primarily attributed to the fact that, in the case of the combined RO-FO method, hydraulic pressure was applied for the determination of A and B, which possibly altered the properties of the membrane as well as introduced errors in the comparison to the pure-FO method described above. Although this method could clearly provide very accurate estimates, it is tedious and, as noted by Huang et al. [70], the pre-requirements of using this method are often too difficult to meet under typical lab conditions. A reasonable compromise between the pressurized RO/FO method and this pure-FO method is offered by the “ultra-low-pressure RO–FO” method described in Section 2.1.1b.

### 2.2. Analytical Approach-Based “Direct” Techniques

#### 2.2.1. Using Tortuosity Calculation Models

Kim et al. [73] proposed a direct analytical approach to estimate the S-values of FO membranes. This is in contrast with the previously discussed methods where the S-value was calculated *indirectly* from the $J_{W}$ and $J_{S}$ expressions. This method involves estimating the tortuosity, porosity, and thickness individually and estimating the S-value thereof. The thickness and porosity measurements were performed using conventional techniques, such as scanning electron microscopy (SEM) and gravimetric measurements. A fractal-theory-based model that relates tortuosity and porosity was used to determine the tortuosity of the porous support [73]. The S-value was calculated using these tortuosity, porosity, and thickness values. However, comparison with the so-called indirect methods revealed significant (~*3–5X*) deviations in the S-values. Although experimental methods naturally involve some degree of measurement errors, empirical models like the ones developed by Kim et al., also involve various assumptions and approximations, which could lead to significant errors in estimation. Without validation, it is, therefore, uncertain whether this method could be an alternative to previously discussed methods.

#### 2.2.2. Using Mercury Intrusion Porosimetry and X-ray Microscopy

Manickam et al. [74] described two analytical approaches to calculate the S-value of osmotic membranes: (i) mercury intrusion porosimetry (MIP) coupled with a micrometer, and (ii) micro X-ray microscopy (Micro-XRM). For the first approach, the authors calculated the average porosity and thickness with the help of MIP coupled with a micrometer. For the second approach, Micro-XRM was used to calculate average thickness, porosity, and tortuosity. In comparison with the indirect approach (described in Section 2.1.1a), their S-values for both the MIP and Micro-XRM techniques were 1–2 orders of magnitude lower. The low values were attributed to mass transfer limitations, and poor wetting of the hydrophobic materials, as well as the effects of the hydrodynamic conditions. In summary, significant uncertainty exists in the determination of S-values using such direct analytical methods.

## 3. Modification Strategies of FO Membrane Substrates with *S*-Values

Various research groups have developed a range of support layer fabrication strategies, the most notable of which will be discussed in this article. Common methods to prepare the FO support layer are solvent casting, electrospinning, and hollow fiber spinning. In many cases, the addition of nanomaterials has been found to enhance the transport and mechanical properties of FO membranes, as will be described in the following sections.

A review of FO membranes’ fabrication revealed significant variations in the membrane testing conditions (especially feed/draw solution composition and pressure) used by different research groups, which creates difficulties in comparing these membranes. The properties of water flux, solute flux, etc., are especially sensitive to such testing conditions. The S-value, on the other hand, is essentially an intrinsic membrane property, making it less susceptible to varied test conditions. It should be noted, though, that a variation of S-value with feed solution salinity levels has been documented for other osmotic processes, such as osmotically assisted reverse osmosis (OSRO) [78], and pressure retarded osmosis (PRO) [79], but so far, has not been reported for FO membranes.

### 3.1. Electrospinning

**Figure 3:**
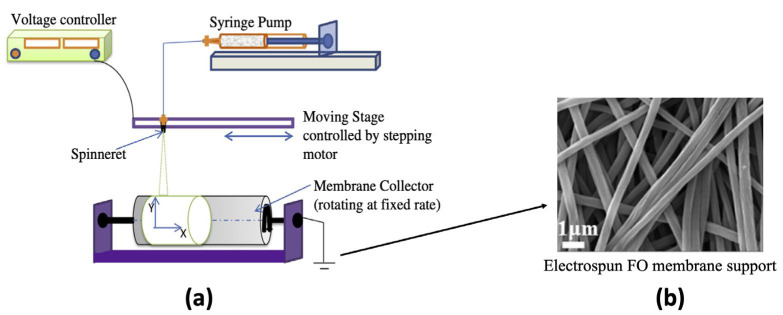
(**a**) Schematic diagram of electrospinning method (reproduced from Tian et al. [85] with permission from Elsevier); (**b**) FO support layer fabricated using electrospinning technique (reproduced from Han et al. [86] with permission from Elsevier).

Electrospinning produces continuous polymer nanofibers with reasonable mechanical strengths, [80,81,82]. The nanofiber diameters typically range from nano to micrometers [81,82]. The electrospinning technique involves extrusion of the polymer solution via a spinneret under a specific voltage causing the formation of a fiber jet stream, which gets collected on a collector drum as highly porous nanofibers (Figure 3). With large surface areas with interconnected pores, electrospun nanofiber support-based FO membranes provide significant advantages over conventional solvent casting process [83]. The electrospinning process provides a high degree of tunability, where the surface chemistry, mechanical properties, and the porosity of the support can be tuned simply by modifying parameters such as voltage, feed rate, collector-feed distance, and nozzle diameter [84]. Careful selection of different polymers and post-treatment strategies offer additional tuning parameters. This section of the article reviews the electrospinning strategies used by different authors, and Table 2 details the performance of some of the high-performance electrospun FO membranes.

#### 3.1.1. Electrospinning without the Incorporation of Nanomaterials

A high-performance TFC-FO membrane using a polyacrylonitrile (PAN) nanofiber substrate as the support layer has been described by Han et al. [86]. Their work highlighted the importance of creating aligned (degree of alignment 92%) vs. randomly oriented (degree of alignment ~28%) nanofibrous structures by tuning the speed (1500 and 500 rpm). No significant change in apparent porosity was observed for both aligned and randomly oriented supports (~84% porosity in case of aligned vs. ~81% porosity in case of randomly oriented); however, the former case presumably leads to better pore interconnectivity. The presence of interconnected pores in the case of the aligned structure imparted higher hydrophilicity to the substrate (~55°) compared to the randomly oriented one (~65°). The combination of porosity and hydrophilicity resulted in a very low S-value (~86 μm) in the case of the aligned FO membrane substrate vs. the randomly oriented FO substrate (~163 μm), and in fact, *this is the lowest* S*-value that has been reported for electrospun supports*. When tested under FO conditions with 1 M NaCl as the draw and DI water as the feed, these aligned electrospun PAN membranes showed significantly higher fluxes (~51 LMH) vs. randomly oriented PAN-supported membranes (~25 LMH).

Another notable example was the development of an FO support layer by Park et al. [87], where the authors developed polyvinyl alcohol (PVA)-coated electrospun polyvinylidene fluoride (PVDF) membranes. This work utilized the easy processability and robustness of PVDF membranes and the hydrophilic functionality was imparted by the PVA coating. The dip-coating of polyvinyl alcohol (PVA) followed by crosslinking with glutaraldehyde, enhanced the hydrophilicity of the electrospun PVDF membrane, as was reflected in a ~*2X* drop in contact angle from 138° to 66°. The low contact angle and the high porosity (~75% porosity) of this electrospun material resulted in a low S-value of 154 µm as well as low ICP effects. When tested under identical experimental conditions, the commercial HTI-CTA FO membrane showed an S-value of ~690 μm. The water flux (~25 LMH) obtained through these membranes was also higher than that of a commercial HTI-CTA membrane (~7 LMH) when tested with 0.5 M NaCl as the draw and DI water as the feed.

**Figure 4:**
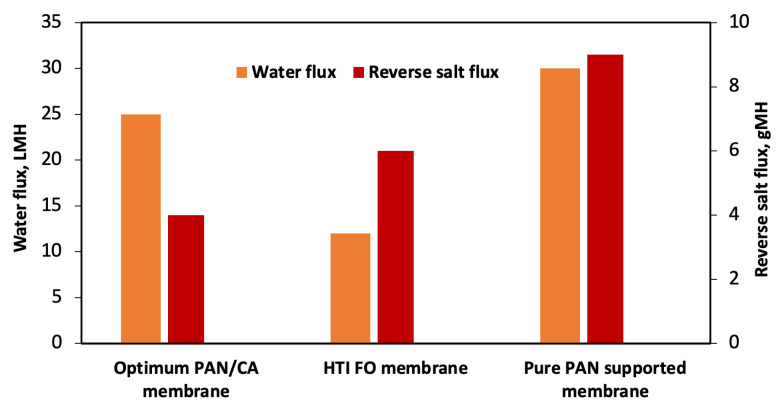
Water and reverse salt flux of an optimum CA-supported PAN membrane, commercial HTI flat sheet FO membrane and pure PAN-supported FO membrane at 25 °C with a 1.5 M NaCl draw and DI water feed solution. Figure drawn using average values taken from Bui et al. [88].

Cellulose acetate (CA) membranes are the “workhorse” materials for traditional pressure-driven separation processes; however, processing these materials into electrospun FO membranes is challenging. Bui et al. [88] overcame this processability issue by blending CA with polyacrylonitrile (PAN) in varying amounts and with an optimum mixing ratio, the composite membrane had a *2X* higher water flux (~25 LMH) than the commercial HTI FO membrane (~12 LMH) under FO mode with a 1.5 M NaCl draw and DI water feed solution (Figure 4). An optimum mixing ratio ensured low hydrophilicity, reasonable mechanical strength, and attractive separation performance. In fact, the performance of the composite membrane exceeded that of the pure PAN and CA membranes. The optimized PAN/CA composite membrane had significantly lower (~*3X*) reverse salt flux, compared to the pure PAN-supported FO membrane (Figure 4). Although pure PAN-supported FO membrane had a high water flux (~30 LMH), the usage of this membrane for FO applications is not viable because of its high reverse salt flux (Figure 4). The S-value of the PAN/CA electrospun membrane (311 μm) was also significantly lower than commercial HTI FO membranes (~578 μm).

Polyamides (nylon—6,6), which are state-of-the-art membrane materials for NF/RO membranes, can also be electrospun into high performance FO membrane supports, as demonstrated by Huang et al. [89]. In addition to electrospun support layer fabrication, this paper explored variations in the interfacial polymerization process of polyamide formation, however, such discussions related to the selective layer formation are outside the defined scope of this article. This support layer was extremely hydrophilic with a contact angle of 38°. The estimated S-value of the support was around 190 µm; ~*2X* lower than commercial HTI membranes, and this low S-value resulted in higher water fluxes than commercial HTI cellulose acetate membranes. The water flux of the optimized nylon-based FO membrane was *~2X* higher than the commercial HTI FO membrane under FO mode with 1 M NaCl as the draw and DI water as the feed solution. In this work, the authors provided some critical insights regarding the mechanical properties of these membranes. It was noted that these membranes had reasonably good mechanical properties under dry conditions—comparable to most phase inversion supports. However, when tested under “wet conditions”, a significant reduction in mechanical property was observed, which was attributed to the excessive swelling of nylon-6,6. Such swelling behaviors are expected for most hydrophilic nanofiber supports. This work effectively highlighted the importance of evaluating FO membrane characteristics under wet conditions.

**Table 2:** Comparison in performance of different FO membranes with support layers fabricated by the electrospinning method. For better understanding, we designated the different methods used to calculate the intrinsic parameters (refer to discussion in Section 2)—method #1 is pressurized RO-FO (Section 2.1.1a); method #2 is ultra-low-pressure RO-FO (Section 2.1.1b); and method #3 is single stage FO (Section 2.1.2).

Support Layer Materials	FO Draw Solution	$\mathrm{Water}\;\mathrm{Flux},\;J_{W}\;(\mathrm{LMH}){\;}^{a}$	Water Permeability, A (LMH/bar) ^b^	Method to Calculate A	*S*-Value (μm)	References
PVDF/PVA	0.5 M NaCl	24.8	1.94	#3	154	[87]
PAN	1.0 M NaCl	50.7	3.23	#2	86	[86]
PAN	2.0 M NaCl	41.0	1.47	#3	168	[90]
PSf/PAN	1.0 M NaCl	38.3 ^c^	3.68	#1	340	[91]
PVDF	1.0 M NaCl	22.0	1.28	#2	193	[70]
PET/PSf	1.0 M NaCl	13.0	1.13	#1	651	[92]
Nylon 6, 6	1.0 M NaCl	21.0	1.66	#1	190	[89]
PVDF	1.0 M NaCl	28.0	3.15	#1	325	[85]
PAN/CA	1.5 M NaCl	25.0	1.79	#1	311	[88]
CA/PVDF	0.5 M NaCl	31.3	2.79	#3	190	[93]

^a^ The operating temperature was 25 ± 5 °C and DI water was used as the feed solution. The experiments were performed in AL-FS mode. ^b^ Operating temperature used in the RO experiments = 25 ± 5 °C. ^c^ FO experiments were performed in AL-DS mode and DI water was used as the feed solution.

As is evident from Table 2, different research groups have used varying testing conditions for these membranes, which makes it difficult to compare their intrinsic water and salt permeabilities. It is, however, possible to focus on reduced ICP effects rendered by improved support development and the S-value serves as a great tool to quantitatively assess the support efficacy.

#### 3.1.2. Electrospinning with the Incorporation of Nanomaterials

The addition of nanomaterials to electrospun supports helps to enhance the mechanical stability and also contributes to improved hydrophilicity and porosity of the support layer [94]. Metal oxide and carbon-based nanomaterials have been widely used with polymers in the support layer to provide stability, low roughness, and good antifouling properties [95,96]. This section reviews the role of a nanomaterial-embedded electrospun FO support in reducing ICP.

Carbon-based nanomaterials are predominantly used for this purpose to increase the hydrophilicity and porosity of FO membranes [95,97]. For example, as shown in the work by Tian et al. [94], functionalized multi-walled carbon nanotubes (f-CNTs) integrated into a polyetherimide (PEI) support layer exhibited an 18% increase in porosity and 53% increase in tensile modulus in comparison with pure PEI electrospun substrates. The inclusion of these nanomaterials also improved the membrane surface’s hydrophilicity. This led to an overall reduction in the S-value with respect to the pure PEI substrate (~674 µm vs. 310 µm). Under FO conditions with a 1 M NaCl draw and DI water feed solution, FO membranes with nanomaterial-incorporated supports achieved ~*3X* higher water flux (~33 LMH) compared to the commercial HTI-CTA FO membrane (~10 LMH).

### 3.2. Solvent Casting

**Figure 5:**
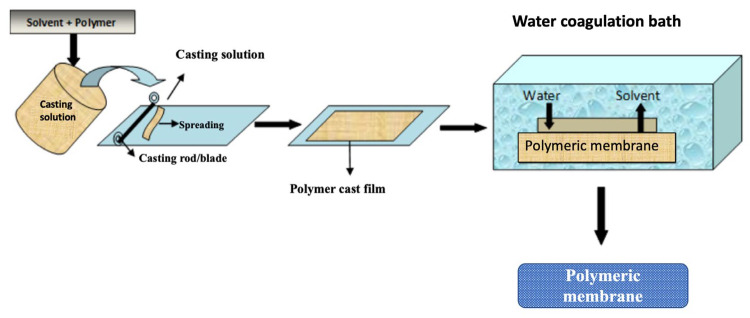
Solvent Casting Method for the preparation of polymeric membranes (reproduced from Zahid et al. [103]).

Solvent casting involves dissolving one or more polymers in specific solvent and casting this onto a support, followed by immersion in a non-solvent (primarily water) to facilitate phase separation (Figure 5). Although solvent casting has traditionally been used to create porous microfiltration/ultrafiltration membranes, it can also be used to create porous FO membrane supports with low ICP effects via variation of the casting parameters. The formation of the porous structure and its characteristics is influenced by the material used for membrane formation. In addition to different polymers, nanomaterials are often added to the polymer solution to tune the overall porosity. Similar to pressure-driven UF/MF processes, polysulfone and sulfonated copolymers are most commonly used to design the solvent-cast FO membrane support layers [98,99,100,101,102]. In most cases, the selective layer is designed through the interfacial polymerization of m-phenylene diamine (MPD) and trimesoyl chloride (TMC). In this section, we will discuss a few examples of support layer design through the solvent casting method with or without incorporating nanomaterials; Table 3 and Table 4 list the performance of such FO membranes.

#### 3.2.1. Solvent Casting without the Incorporation of Nanomaterials

Blending pure polysulfone with other sulfonated polymers has been commonly adopted by multiple researchers, since they ensure compatibility between the two polymers and help to engineer the support layer’s porous structure. Zhang et al. [98] described a support layer that blended polysulfone (PSf) with optimum amounts of a disulfonated poly(arylene ether sulfone) hydrophilic-hydrophobic multiblock copolymer. It was hypothesized by the authors that the presence of such a copolymer can delay demixing during the phase inversion process, which is useful for achieving a sponge-like macrovoid-free structure. Such a structure is preferred for better FO performance and mechanical stability. The pristine PSf substrate exhibited a significant number of macrovoids because of the instantaneous demixing during phase inversion. Although a slight decline in tensile modulus was observed in the case of the blended membrane, the authors showed that the latter possesses enough flexibility that it can be rolled or processed into useful membrane formats. Blending with this hydrophilic-hydrophobic block copolymer resulted in an FO membrane with a significantly lower S-value (186 μm) compared to the pure PSf membrane (495 μm). This can be primarily attributed to a slightly high porosity and significantly lower contact angle (57°) than pristine PSf (81°). Slight thickness reductions were also observed for the blended support, but the cause behind this was not discussed in the paper. The water flux of the modified membrane (~41 LMH) was ~*2X* higher compared to the pure PSf membrane (~20 LMH) when they were tested under FO mode with a 2 M NaCl draw and DI water feed solution. The authors further evaluated the performance of these membranes with 3.5 wt.% NaCl as the feed (sea water concentration) and 2 M NaCl as the draw solution and, even in presence of apparent ICP effects, the flux obtained through these membranes was one of the highest (>18 LMH) reported under these conditions.

**Figure 6:**
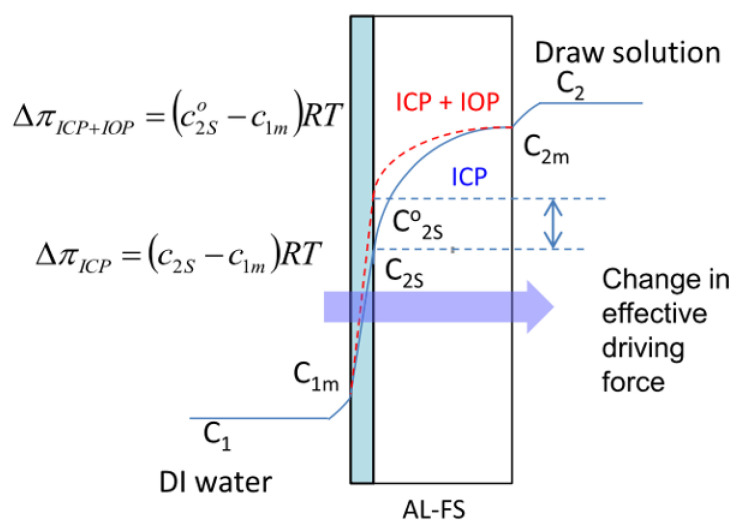
ICP profile of a typical FO membrane with (dotted lines) and without (solid lines) an IOP in AL-FS mode. Concentration on the feed side, draw solution side, selective layer–feed water interface and selective layer–support layer side were denoted using subscripts 1, 2, m and s, respectively. Concentrations in the presence of IOP are denoted using superscript “o” (reproduced from Zhou et al. [104] with permission from Elsevier).

A similar approach to blend two or more polymers for engineering the porous structure was adopted by Zhou et al. [104], where a combination of polysulfone (PSf) and sulfonated poly(phenylene oxide) (SPPO) was implemented. This work uniquely utilizes the high ion-exchange capacity of SPPO to generate an internal osmotic pressure (IOP) via immobilization of Na^+^ ions present in the draw solution. To describe briefly, the PSf/SPPO blended membrane was washed with dilute hydrochloric acid, which led to the formation of H^+^ ions. These H^+^ ions act as the ion exchange spots, which serve to partially immobilize the Na^+^ present in the 2 M NaCl draw solution. As illustrated in Figure 6 below, this partial immobilization appears to create an increased osmotic pressure difference, which translates to a higher flux through the membranes by countering the ICP effect. In addition to this unusual IOP effect, the addition of SPPO also enhanced the hydrophilicity (60–63° instead of 73° for pure PSf) and the support layer’s porosity—both of which factors resulted in a lower S-value compared to the pristine PSf-based membranes. It must be noted though, that the S-value for the PSf membrane noted in this study was exceptionally high (>3500 μm), but in any case, the comparison under similar testing conditions showed a *10X* reduction in S-value for the blended membrane. A low S-value resulted in a ~*3X* times higher water flux (~39 LMH) compared to the commercial HTI-flat sheet FO membrane (~13 LMH) and reduced the reverse salt flux (~11 gMH to ~6 gMH) using a 2 M NaCl draw and DI water as the feed. Interestingly, the IOP effect enabled the PSf/SPPO blended membrane to surpass the performance of many commercial counterparts when the membrane was tested for a sea water desalination case, with a 3.5 wt.% NaCl (0.6 M) solution as the feed and 2 M NaCl as the draw. As expected, the absolute values of the fluxes were low due to excessive concentration polarization, but the water flux of the blended membrane was significantly higher (~20 to ~25 LMH) in comparison to commercial membranes (~4 to ~10 LMH). These membranes, as well as the one described earlier, are among the few demonstrations where realistic concentrations were used to evaluate the membrane performance and more such experiments should be reported going forward. In fact, since produced water sources could have even higher salinity levels than seawater, it is recommended that feed concentrations beyond 3.5 wt.% NaCl be used for membrane testing.

**Figure 7:**
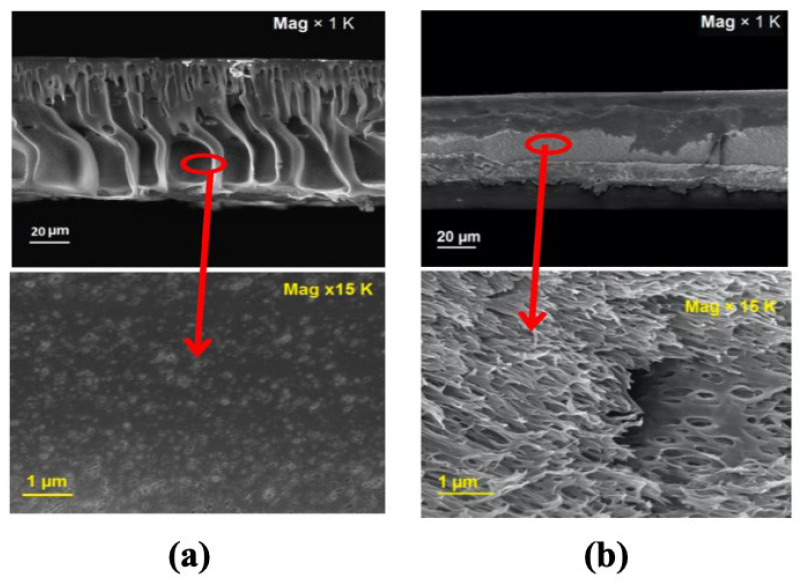
SEM images of the membrane substrate containing: (**a**) Pure PES; and (**b**) PES/SPES (50 wt.% SPES) (reproduced from Sahebi et al. [99] with permission from Elsevier).

Additional demonstrations of the blended sulfonated polymer approach have been described by Han et al. [105] and Sahebi et al. [99]. Han et al. [105] fabricated the support layer using super hydrophilic sulfonated poly(ether ketone) (SPEK) and polysulfone (PSf) polymers and this membrane composition resulted in one of the lowest S-values (107 μm) reported for solvent-cast FO supports. This work shows that precisely controlling the amount of SPEK in the casting mixture is key to achieving the desired morphology as well as the critically important properties, such as high porosity and high hydrophilicity. With a 50% SPEK addition, the water flux significantly increased (35 LMH) compared to pristine PSf (23 LMH) when tested with 2 M NaCl as the draw and DI water as the feed. Similar to some of the previous articles, lower magnitudes of water flux (17 LMH) were observed with simulated seawater desalination tests where 3.5 wt.% NaCl solution was used as the feed and 2 M NaCl as the draw solution. This work highlighted the importance of developing sponge-like pores in the support layer to reduce the impact of ICP during FO operations. Similar phenomena were observed by Sahebi et al. [99], where a combination of polyethersulfone (PES) and sulfonated polyethersulfone (SPES) was used to design the support layer. Support layers with optimum sulfonation degrees facilitate creating sponge-like porous substrates with lower thicknesses, while also avoiding macrovoid formation, unlike pure PES membranes (Figure 7). Both thickness reduction and sponge-like porous formation were the effects of the delayed phase separation induced by sulfonation. In addition, a drastic decrease in the contact angle for the sulfonated PES (~20°) was observed compared to pure PES (77°). Increasing the SPES content beyond a certain point, however, leads to a decrease in the substrate’s mechanical strength. Nevertheless, with an optimum degree of sulfonation, the overall S-value of the substrate was reduced to 245 µm from 1096 µm in the unmodified substrate. This was mainly because of the high hydrophilicity of the SPES substrate as well as the low overall thickness of the modified membranes (65 μm instead of 80 μm in pure PES). The blended sulfonated FO membrane exhibited a water flux of ~35 LMH, which is ~*2X* pure PSf (~17 LMH) and ~*2.5X* commercial HTI (~13 LMH) FO membrane using a 2 M NaCl draw and DI water as the feed.

Zheng et al. [106] described an approach where sulfonated polysulfone (SPSU) was blended with polyvinyl chloride (PVC) to lower the S-value and reduce the ICP in the resulting FO membrane. Increasing the SPSU content up to 10% with PVC resulted in an increase in porosity with respect to pure PVC (increased from 81% to 90%), as well as an increase in the hydrophilicity in which the contact angle decreased from ~85° to ~75°. These factors, along with good pore interconnectivity, resulted in a considerably lower S-value. An addition of 10% SPSU resulted in a *10X* reduction in S-value, from ~2668 μm in the pure PVC membrane to ~286 μm in the 10% SPSU-blended PVC membrane, when tested with 1 M NaCl as the draw solution and DI water as the feed solution. The blending of SPSU also influenced the subsequent looser polyamide layer deposition and led to higher A values in the overall membrane, along with higher salt permeability values. When compared with other sulfonated membranes, these PVC/SPSU blended membranes showed competitive and, in some cases, superior performance. In fact, with the addition of 10% SPSU with PVC, the water flux of the FO membrane increased significantly (~28 LMH for 10% SPSU/PVC and ~4 LMH for pure PVC) when tested under a 1 M NaCl draw and DI water feed solution.

**Table 3:** Comparison in performance of different FO membranes with support layers fabricated by the solvent casting method without the incorporation of nanomaterials. For better understanding, we designated the different methods used to calculate the intrinsic parameters (refer to the discussion in Section 2)—method #1 is pressurized RO-FO (Section 2.1.1a); method #2 is ultra-low-pressure RO-FO (Section 2.1.1b); and method #3 is single stage FO (Section 2.1.2).

Support Layer Materials	FO Draw Solution	$\mathrm{Water}\;\mathrm{Flux},\;J_{W}\;(\mathrm{LMH}){\;}^{a}$	Water Permeability, A (LMH/bar) ^b^	Method to Calculate *A*	*S*-Value (μm)	References
PSf/PSf-g-PDMA	1.0 M NaCl	16.4 ^d^	2.05	#1	546	[107]
SPSU/PVC	1.0 M NaCl	27.9 ^d^	2.80 ^c^	#1	286	[106]
PVDF/PFSA	1.0 M NaCl	27.0 ^d^	2.97	#2	335	[108]
PES/SPSU	2.0 M NaCl	26.0	0.77	#1	238	[101]
PSf/BPSH100-BPS0 ^f^	2.0 M NaCl	40.9	1.57 ^c^	#1	186	[98]
PSf/SPPO	2.0 M NaCl	39.0	3.55	#1	293	[104]
PSf/SPEK	2.0 M NaCl	35.0	0.75 ^c^	#1	107	[105]
sPPSU	2.0 M NaCl ^e^	17.5	3.70 ^c^	#2	256	[109]
PES/PESU-co-sPPSU	2.0 M NaCl	21.0	0.73 ^c^	#1	324	[110]
PES/SPES	2.0 M NaCl	35.1	2.90 ^c^	#1	245	[99]
PES/NaHCO_3_/PEG	1.0 M NaCl	26.6	2.13	#1	257	[111]

^a^ The operating temperature was 25 ± 5 °C and DI water was used as the feed solution. The experiments were performed in AL-FS mode. ^b^ Operating temperature used in the RO experiments = 25 ± 5 °C. ^c^ Operating temperature for the RO experiments was not reported. ^d^ Operating temperature for the FO experiments was not reported. ^e^ Feed solution concentration = 3.5 wt.% NaCl. ^f^ BPSH100-BPS0 = disulfonated poly(arylene ether sulfone) multiblock copolymer.

#### 3.2.2. Solvent Casting with the Incorporation of Nanomaterials

**Figure 8:**
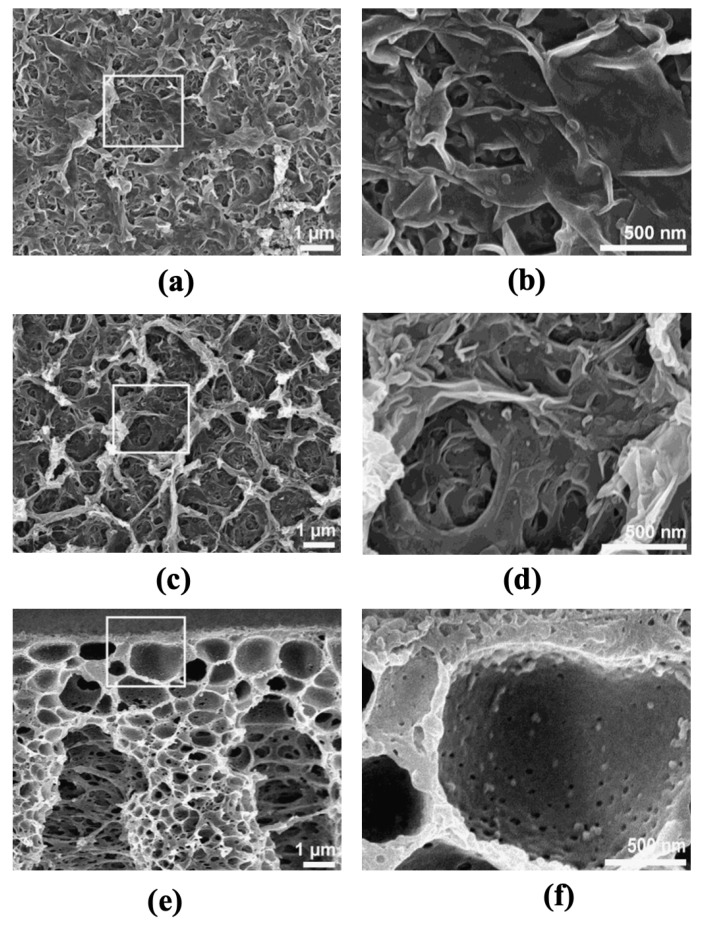
SEM surface image of (**a**,**b**) pure PSf-supported FO and (**c**,**d**) optimum PSf/NaY-supported FO membrane. (**e**,**f**) SEM cross-sectional images of optimum PSf/NaY-supported FO membrane (reproduced from Ma et al. [112] with permission from Elsevier).

**Figure 9:**
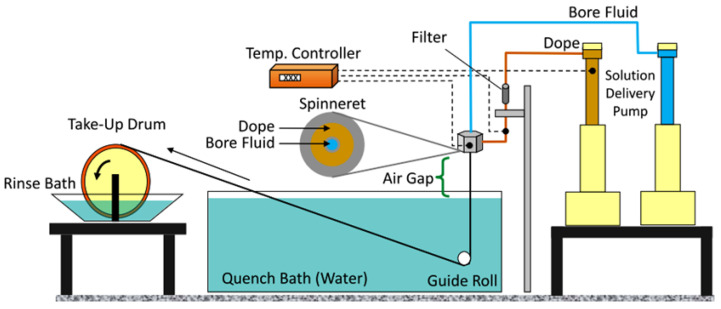
Hollow fiber membrane spinning setup (reproduced from Chen et al. [130] with permission from Elsevier).

Similar to the case of electrospinning, the inclusion of nanomaterials helps to tune the porosity and pore interconnectivity of the solvent-cast FO membrane supports and, in turn, reduce the S-value. A notable example of such nanomaterial-embedded solvent cast supports has been described by Ma et al. [112], where the PSf-based FO support layer was modified via the inclusion of porous zeolite nanoparticles (NaY). Introduction of NaY reduced the S-value of the PSf substrate by a factor of three (~340 μm vs. 960 μm), which is surprising since no significant changes occurred in terms of thickness, porosity, and hydrophilicity on zeolite incorporation. We speculate that this reduction was primarily due to better pore interconnectivity attained in the zeolite-incorporated polysulfone membrane, although the SEM images provided are not conclusive enough (Figure 8). A cross-sectional SEM image of pure PSf would have been more helpful, as this would enable a direct comparison with the cross-sectional image of the PSf/NaY-supported membrane, as shown in Figure 9e,f. Nevertheless, this lower S-value translated into a higher water flux (~40 LMH) than the pure PSf substrate (~15 LMH), under FO mode with a 2 M NaCl draw and DI water feed solution. The authors also conducted an experiment using different saline feed solutions by varying the salt concentrations (0.1 M to 0.5 M) and observed a low water flux because of a higher ICP effect in the saline feed solution. However, the water flux of the composite PSf/NaY membrane still showed superior performance compared to the pure PSf membrane. In fact, testing with a 0.5 M NaCl feed and 2 M NaCl draw solution, the PSf/NaY modified membrane showed a *~3X* higher flux in comparison with the pure PSf membrane.

A similar concept was described by Emadzadeh et al. [113], where titanium dioxide (TiO_2_) nanoparticles were incorporated within a polysulfone (PSf) support layer. The delayed phase separation that occurs due to the addition of nanomaterials was believed to contribute to higher porosity in the nanocomposite substrate (82%) in comparison to the pure PSf substrate (68%). In addition, higher hydrophilicity (51°) was found in the TiO_2_/PSf substrate compared to the PSf substrate (67°). The combination of increased porosity and hydrophilicity caused an overall reduction in the S-value from 910 µm to 390 µm for the optimized membrane. As a result of this decreased S-value, the reported water flux for the optimized membrane was ~33 LMH, *~2X* higher than the lab-made pure PSf-supported FO membrane (~14 LMH) under FO mode with a 2 M NaCl draw and DI water feed solution. Similar observations were documented by Ghanbari et al. [114], where significant (~*2.5X*) reductions in S-values were achieved via the incorporation of halloysite nanotubes (HNT), which are aluminosilicate materials with a chemical composition of (Al_2_Si_2_O_5_(OH)_4_·2H_2_O). A considerable improvement in the substrate hydrophilicity and surface mean pore size was hypothesized to primarily contribute to the low S-value, as was the case in other articles described above. These results underscore the importance of membrane hydrophilicity/wettability for FO applications, as was discussed in Section 1.1 in the context of the paper by McCutcheon et al. [36].

Metal organic frameworks (MOFs), which have been extensively utilized in the area of gas separations, have also been used to make nanomaterial-embedded FO membrane supports [115,116]. In a recent work, Ma et al. [115] described the use of UiO-66 MOFs within a PSf support. The UiO-66 structure consists of [Zr_6_O_4_(OH)_4_]-based metal clusters and 1,4—benzo dicarboxylic acid organic linkers, and unlike many other MOFs, they are stable in water. No significant change in hydrophilicity, overall porosity and substrate thickness were observed, and in fact, the mean effective pore size and the molecular weight cut off (MWCO) of the pristine membrane were reduced due to the addition of UiO-66. Despite these facts, a *1.5X* reduction in S-value was achieved—~351 μm in the PSf/UiO-66 membrane vs.~528 μm for the PSf-supported FO membrane. The UiO-66 modified FO membrane also exhibited a *1.5X* higher water flux (~25 LMH) compared to the pristine PSf-supported FO membrane (~16 LMH) under FO mode with a 1 M NaCl draw and DI water feed solution. We hypothesize that the UiO-66 MOFs provide additional channels for water and salt diffusion through the support, which are not reflected in the porosity measurements. Intrinsic diffusion coefficient measurements of pure PSf, pure UiO-66, and the composite membrane, could be useful to verify this hypothesis.

Based on the discussion of Section 3.1 and Section 3.2, it is evident that electrospinning, in most cases, offers superior structural parameters and FO properties over solvent casting/phase inversion. The membrane prepared from Han et al. [86] through electrospinning had the best S-value (~86 μm) among all the membranes that are reviewed for this paper. Inclusion of nanomaterials helps to improve FO membrane performance by improving surface properties, such as hydrophilicity, porosity, and mechanical strength, as is evident from Section 3.1.1 and Section 3.2.2.

**Table 4:** Comparison in performance of different FO membranes with support layers fabricated by the solvent casting method with the incorporation of nanomaterials. For better understanding, we designated the different methods used to calculate the intrinsic parameters (refer to the discussion in Section 2)—method #1 is pressurized RO-FO (Section 2.1.1a); method #2 is ultra-low-pressure RO-FO (Section 2.1.1b); and method #3 is single stage FO (Section 2.1.2).

Support Layer Materials	FO Draw Solution	$\mathrm{Water}\;\mathrm{Flux},\;J_{W}\;(\mathrm{LMH}){\;}^{a}$	Water Permeability, A (LMH/bar) ^b^	Method to Calculate A	*S*-Value (μm)	References
GO/PSf	0.5 M NaCl	19.8	1.76	#1	191	[97]
Al_2_O_3_/PSf	1.0 M NaCl	27.6	8.43	#1	1028	[117]
Zn_2_GeO_4_/PES	1.0 M NaCl	15.0 ^d^	2.47 ^c^	#1	540	[96]
INTs/PSf ^f^	1.0 M NaCl	7.5	3.03 ^c^	#1	2090	[118]
TiO_2_/PSf	2.0 M NaCl	33.0	2.63 ^c^	#1	390	[113]
HNT/PSf ^g^	2.0 M NaCl ^e^	27.7	2.00 ^c^	#1	370	[114]
NaY(zeolite)/PSf	2.0 M NaCl	40.0 ^d^	3.30	#1	340	[112]
PSf/UiO-66 ^h^	1.0 M NaCl	24.5 ^d^	3.31 ^c^	#2	351	[115]
GP/PSf ^i^	1.0 M NaCl	15.6	3.12	#1	711	[119]
MWCNT/PES ^j^	2.0 M glucose ^e^	12.0 ^d,k^	2.31 ^c^	#1	2042	[95]

^a^ The operating temperature was 25 ± 5 °C and DI water was used as the feed solution. The experiments were performed in AL-FS mode. ^b^ Operating temperature used in the RO experiments = 25 ± 5 °C. ^c^ Operating temperature for the RO was not recorded. ^d^ Operating temperature for the FO was not recorded. ^e^ Feed solution concentration = 10 mM NaCl. ^f^ INT = Imogolite nanotubes containing Al, Si and OH with a ratio of 2:1:4. ^g^ HNT = Halloysite nanotubes with a chemical composition of Al_2_Si_2_O_5_(OH)_4_·2H_2_O. ^h^ UiO-66 nano crystals were synthesized using 1,4-benzenedicarboxylic acid (BDC), zirconium chloride (ZrCl4), N,N-dimethylformamide (DMF), and acetic acid (AA). ^i^ GP = Graphene oxide-graft-poly(2- hydroxy ethyl methacrylate) nanoparticles. ^j^ MWCNT = Multi-walled carbon nanotubes. ^k^ The experiment was performed in AL-DS mode.

### 3.3. Hollow Fiber FO Membrane Support

Unlike pressure-driven desalination processes where spiral wound membranes are state-of-the-art, hollow fiber formats are being considered extensively for FO applications [120,121,122,123,124]. The hollow fiber configuration allows high surface area to volume ratios and, therefore, high overall productivities [125,126], which help to counter flux decline due to polarization effects and fouling. Some of the materials used to fabricate the hollow fiber FO membranes via the traditional dry-jet wet-quench method include polyethersulfone (PES), sulfonated polyphenylenesulfone (sPPSU), and cellulose acetate butyrate (CAB) [127,128,129]. This method involves extruding the polymer “dope” solution along with a bore fluid (mixture of solvent and non-solvent) through an annual die viz., a spinneret followed by quenching in water, eventually to be collected on a drum as hollow fibers (Figure 9) [130]. In this section, we will provide some notable examples of hollow fiber-based FO membranes; Table 5 lists the detailed performance of such membranes.

**Figure 10:**
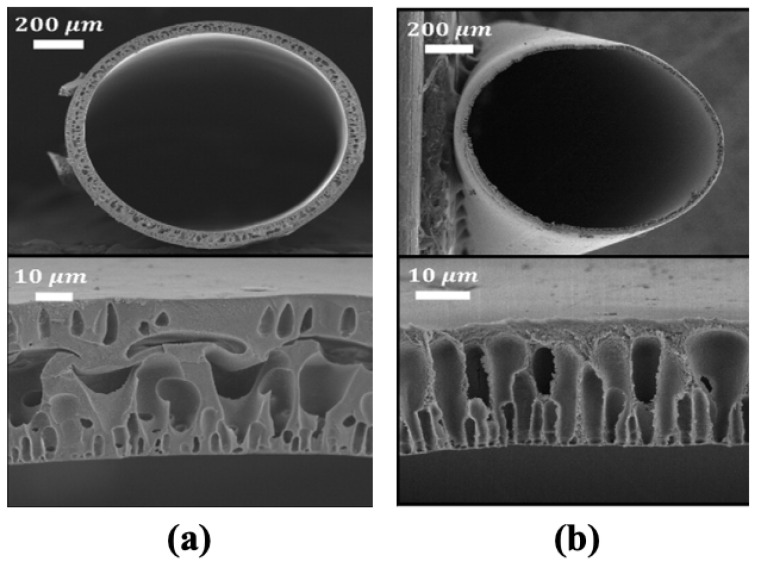
Cross-sectional SEM images of the PAN-supported hollow fiber FO membrane (**a**) PAN-30 with 30 wt.% DMF; and (**b**) PAN-60 with 60 wt.% DMF (reproduced from Ren et al. [131] with permission from Elsevier).

Hollow fiber spinning is well-established for gas and vapor separation processes; however, since the ICP effect is especially pertinent to FO, the spinning procedures must be optimized accordingly. Ren et al. [131] fabricated a polyacrylonitrile (PAN) hollow fiber-supported TFC membrane, and it was demonstrated that the bore fluid composition was an important parameter to develop highly porous supports with low S-values. A N, N-dimethylformamide (DMF)/water mixture was used as the bore fluid and, by varying the ratio of the two solvents, could create a range of porous structures. A sponge-like porous structure was formed (Figure 10a) when the bore fluid concentration (≤30 wt.%—PAN 30) was low, while at higher concentrations (60 wt.%—PAN 60), a finger-like porous structure was obtained (Figure 10b). The reverse salt flux for the PAN-60 substrate-based FO membrane (~19 gMH) was much higher than the PAN-30 (~6 gMH). As PAN substrates were inherently hydrophilic in nature, they provided good wetting properties which resulted in reduced ICP effects. For the PAN-supported hollow fiber membrane, the lowest S-value achieved was about ~305 µm, far lower than the S-value (~533 µm) of commercial HTI-TFC flat sheet membranes. It should be noted, though, that the electrospun PAN supports [86] had a ~*3.5X* lower S-value than the PAN hollow fibers, indicating that the spinning process must be optimized further. The PAN-supported hollow fiber membrane achieved the highest high-water flux of ~25 LMH, which was higher than the HTI-TFC flat sheet FO membrane (~15 LMH) under FO mode with a 1 M NaCl draw and DI water feed solution.

**Figure 11:**
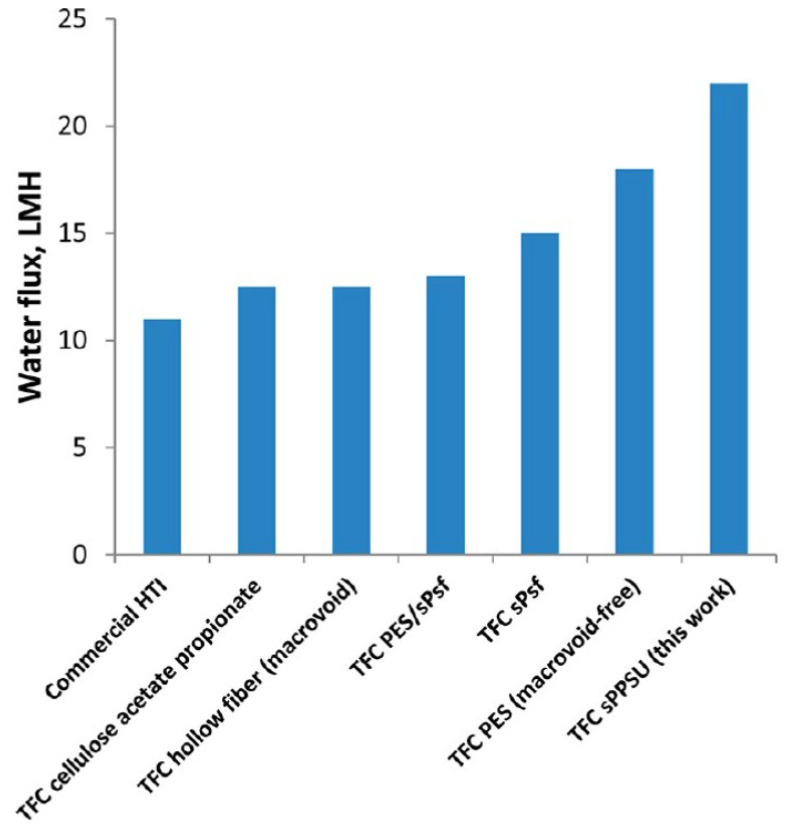
Performance comparison of a TFC-sPPSU (1.5 mole% sPPSU) FO membrane with other FO membranes using a 3.5 wt.% NaCl feed and 2 M NaCl draw solution (reproduced from Zhong et al. [127] with permission from American Chemical Society).

Similar to the cases of electrospinning and solvent casting, sulfonated polymers have been considered a promising approach for hollow fiber-based FO membranes. Zhong et al. [127], for example, used a sulfonated polyphenylenesulfone (sPPSU) dope solution as a substrate and the degree of sulfonation was found to have a direct impact on the porosity and hydrophilicity of the membrane. As expected, the sPPSU-based hollow fiber membranes were highly hydrophilic (contact angle ~69°) compared to the non-sulfonated counterparts (contact angle ~90°). The S-value of the sulfonated PPSU substrate with an optimum degree of sulfonation (~163 µm) was also significantly lower compared to the non-sulfonated PPSU substrate (~746 µm). The sPPSU membrane achieved ~*2X* higher water flux (~23 LMH) compared with the non-sulfonated PPSU hollow fiber membrane (~12 LMH) when they were tested with a 0.5 M NaCl draw and DI water feed solution under FO mode. Similar trends were observed when the membranes were tested with a 3.5 wt.% NaCl feed (model seawater) and 2.0 M NaCl solution as the draw solution. In fact, these membranes showed better performance than a range of other hollow fiber membranes, including commercial HTI membranes, where the water flux of the latter was ~50% of the sPPSU-based membrane (Figure 11).

**Figure 12:**
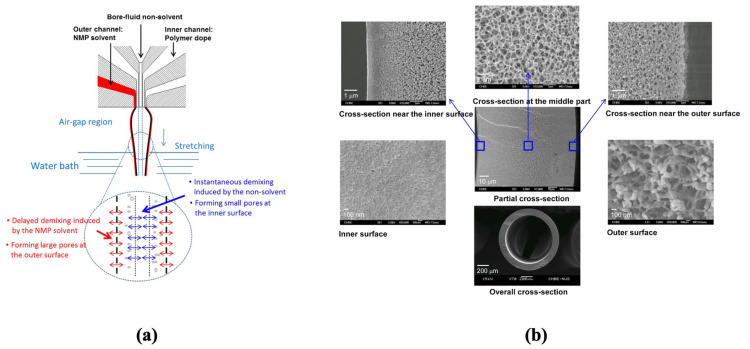
(**a**) Hollow fiber spinning strategies for controlling phase inversion with the help of a co-extruded solvent using a dual layer spinneret. (**b**) SEM surface morphology of PES-supported hollow fiber membrane (reproduced from Sukitpaneenit et al. [128] with permission from the American Chemical Society).

Dual-layer fiber spinning, as has been established by the gas separations membranes community, is an effective tool to engineer the hollow fiber structure without additional post-processing steps. Sukitpaneenit et al. [128] utilized a variation of the technique to form a PES-supported hollow fiber membrane, where along with the dope and bore fluids, an additional third solvent—N-methyl pyrrolidone (NMP)—is co-extruded (Figure 12a). This “*pseudo*-outer layer” of NMP was effective in delaying the phase inversion process, which is key to forming a macrovoid-free sponge-like porous structure. On the other hand, a non-solvent is used as the bore fluid, which causes instantaneous phase inversion and forms a low-porosity inner layer. The bore fluid composition was varied to include three cases: (i) pure water, (ii) 70/30 NMP/water, and (iii) 40/30/30 NMP/water/polyethylene glycol (PEG). The resulting structure was unlike a traditional hollow fiber, which typically has a dense/low-porosity selective layer outside a porous substructure. Instead, this work resulted in a fiber substructure with large, open pores on the outer and middle part with relatively smaller pores in the inner part, as shown in Figure 12b. The selective layer was formed via interfacial polymerization on the lumen side of the hollow fiber membrane. The fiber formed with the NMP/water/PEG mixture as the bore fluid showed the most optimum performance in terms of pure water flux and reverse salt flux. These membranes, when tested with DI water as the feed and 2.0 M NaCl as the draw solution, showed fluxes as high as ~35 LMH and salt leakage of ~10 gMH, and in comparison, the HTI flat sheets had fluxes of ~13 LMH and salt fluxes of ~11 gMH under the same testing conditions. It is our view, though, that comparisons with flat sheet membrane configurations are not entirely appropriate for these hollow fiber membranes. Moreover, the comparison of these membranes with traditional hollow fiber membranes with conventional asymmetric structures, would have helped to justify the complex spinning procedure described in the paper. Nevertheless, it is important to note that this unusual method of hollow fiber spinning resulted in a low S-value of the membrane (~219–261 μm) vs. HTI flat sheets (481 μm) and TFC hollow fiber membranes (550–595 μm). It was further noted, in context of these comparisons, that traditional BW30 RO membranes have an S-value of 40,000 μm, which is ~*100X* higher than most FO membranes discussed here. It is, therefore, abundantly clear that state-of-the-art desalination membranes are not suited for FO applications, and the strategies developed by the FO membrane community to create macrovoid-free porous structures have successfully resulted in an orders of magnitude reduction in S-value.

Hollow fiber spinning has the advantage of high throughput production compared to the earlier discussed techniques. However, on average, it is apparent that electrospun supports show lower S-values than conventionally spun fibers and, to be competitive, hollow fiber spinning must be adapted for FO processes.

**Table 5:** Comparison in performance of different FO membranes with support layers fabricated by hollow fiber spinning technology. For better understanding, we designated the different methods used to calculate the intrinsic parameters (refer to the discussion in Section 2)—method #1 is pressurized RO-FO (Section 2.1.1a); method #2 is ultra-low-pressure RO-FO (Section 2.1.1b); and method #3 is single stage FO (Section 2.1.2).

Support Layer Materials	FO Draw Solution	$\mathrm{Water}\;\mathrm{Flux},\;J_{W}\;(\mathrm{LMH}){\;}^{a}$	Water Permeability, A (LMH/bar) ^c^	Method to Calculate A	*S*-Value (μm)	References
PAN	1.0 M NaCl	24.7	2.15	#2	305	[131]
CAB/PDA	1.0 M NaCl	37.0 ^b^	1.70 ^d^	#2	250	[129]
sPPSU	0.5 M NaCl	22.5	1.99 ^d^	#2	163	[127]
PES	2.0 M NaCl	32.1	1.18 ^d^	#2	219	[128]
PES	1.0 M NaCl	30.2 ^e^	2.26	#3	190	[132]
Polyketone	0.5 M NaCl	40.0 ^b,e^	1.20	#3	250	[133]
PPSU	3.0 M NaCl	13.5 ^g^	2.25	#2	467	[134]
GO/PES	1.0 M NaCl	43.7 ^b,e^	1.27	#1	522	[135]
P84 copolyimide ^f^	1.0 M NaCl	22.0 ^e^	1.22 ^d^	#2	232	[136]
PEI	1.0 M NaCl	38.5	3.66 ^d^	#2	172	[137]

^a^ The operating temperature was 25 ± 5 °C and DI water was used as the feed solution. The experiments were performed in AL-FS mode. ^b^ FO experiments were performed in AL-DS mode and DI water was used as the feed solution. ^c^ Operating temperature used in the RO experiments = 25 ± 5 °C. ^d^ Operating temperature for the RO was not recorded. ^e^ Operating temperature for the FO was not recorded. ^f^ P84 copolyimide = Copolyimide of 3,3′, 4,4′-benzophenone tetra-carboxylic dianhydride with 80% methylphenylene diamine and 20% methylene diamine. ^g^ Feed solution concentration = 0.5 M NaCl.

## 4. Commercial FO Membrane Substrates

**Figure 13:**
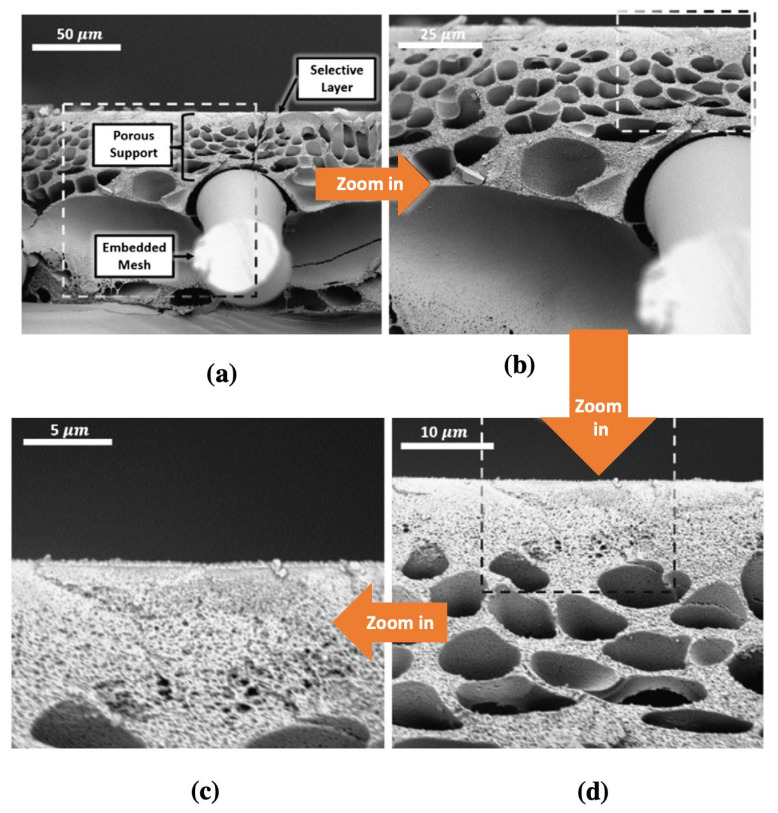
SEM cross-sectional morphologies of the TFC-FO membrane at different magnifications: (**a**) 2000×, (**b**) 2020×, (**c**) 5800×, and (**d**) 11,200× (reproduced from Ren et al. [138] with permission from Elsevier).

Hydration Technology Innovations (HTI) has been the largest supplier of commercial forward osmosis membranes for the last two decades, cellulose acetate-being their preferred material of choice. Most recently, other manufacturers, such as Aquaporin A/S and Oasys Water have also started to produce large-scale forward osmosis membranes. HTI’s new flat sheet TFC-FO membrane, as well as their old cellulose acetate (CA) membrane’s performance, were tested by Ren et al. [138]. In both cases, the underlying polyester fabric provided sufficient mechanical strength and avoided the need for thick support layers. Because of the uniform and defect-free (i.e., devoid of pinholes) morphology of the fabric, the corresponding selective layers of these membranes (TFC-FO and CA membrane) adhered well to the support layer. The TFC-FO membrane was significantly more hydrophilic (contact angle ~14°) than the CA (contact angle 62°) membrane; however, in terms of their S-values, they were pretty similar—HTI-TFC had ~533 µm, whereas the CA membrane had the S-value of ~433 µm. The differences in S-values were attributed to the differences in the thickness, porosity, and tortuosity of these two membranes. The thickness of the TFC support was almost twice the thickness of the CA membrane support (~115 µm and ~50 µm, respectively); however, the TFC membrane’s support had a higher porosity, lower tortuosity, and better wetting capabilities, which translated into higher water and reverse salt fluxes (Figure 13). The pure water fluxes of the CA and TFC membranes were ~8 LMH and ~15 LMH, respectively, and the corresponding reverse salt fluxes were ~1 gMH and ~4 gMH under FO mode with a 1 M NaCl draw and DI water feed solution.

**Figure 14:**
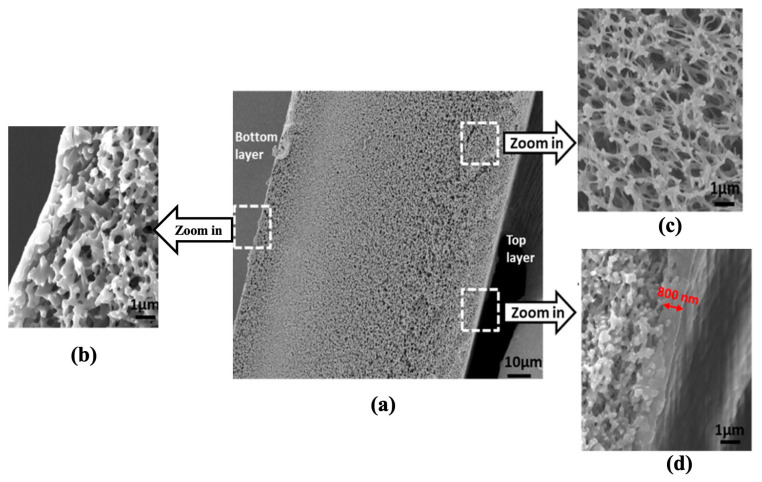
Cross-sectional SEM images of the aquaporin flat-sheet FO membrane at magnifications of (**a**) 750× and (**b**–**d**) 10,000× (reproduced from Xia et al. [139] with permission from the American Chemical Society).

Aquaporin A/S has developed both flat sheet and hollow fiber-based FO membranes. Xia et al. [139] demonstrated the performance of an aquaporin-based flat sheet FO membrane. This TFC membrane consisted of an aquaporin-embedded polyamide selective layer, the support layer material, however, is proprietary. Interestingly, the support layer showed high porosity in the top and bottom sections, while being relatively dense in the middle (Figure 14). The support layer of the FO membrane was highly hydrophilic (contact angle ~35°); however, the S-value was higher (~630 µm). In our view, this could be due to the dense middle zone of the support layer, which could also enhance the ICP effect. The water permeability of the aquaporin FO membrane was ~0.5 LMH/bar, *~3X* lower than the permeability of the HTI-TFC membrane (1.7 LMH/bar). The aquaporin FO membrane had a water flux of ~13 LMH under FO mode with a 1.5 M NaCl draw and DI water feed solution.

**Figure 15:**
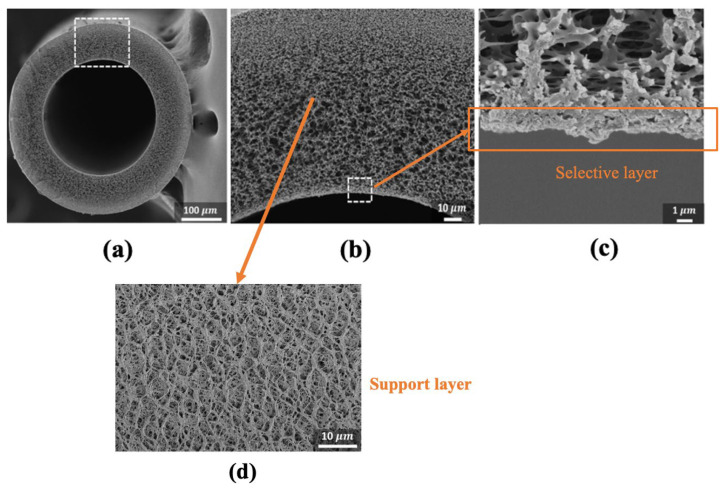
Cross-sectional images of the aquaporin-based hollow fiber membrane at magnifications of (**a**) 170×, (**b**) 800×, (**c**) 5000×, and (**d**) surface of porous support layer at 5000× (reproduced from Ren et al. [140] with permission from Elsevier).

The aquaporin-based commercial hollow membranes were studied extensively by Ren et al. [140]. The fiber support layer had a sponge-like formation with a more open and porous structure and without any macrovoids (Figure 15b,d). A more dense and non-porous selective layer was observed on the inside part of the hollow fiber membrane (Figure 15c). With respect to the flat sheet aquaporin membrane that was discussed earlier by Xia et al. [139], the hollow fiber aquaporin membrane had a comparatively lower S-value (~210 µm). This may be because of the dense middle-zone of the flat sheet aquaporin membrane [139], which was absent in the case of the hollow fiber counterpart [140]. The aquaporin-based FO hollow fiber membrane achieved water and reverse salt fluxes of ~13 LMH and ~1.7 gMH, respectively, in FO mode with a 1 M NaCl draw and DI water feed solution.

Arena et al. [79] studied the Oasys TFC flat sheet membrane in 2015. This TFC-FO membrane contained a support layer that consisted of a polysulfone (PSf) support on top of polyethylene terephthalate (PET). The thickness of this PSf support layer was about ~35 µm. This thin PSf support was the main reason for achieving a low S-value (~483 µm) with reduced ICP. The overall surface porosity of the support layer was approximately 65%. Although the membrane had a more hydrophilic selective layer (contact angle ~40°), its support layer was relatively hydrophobic (contact angle >90°). This FO membrane achieved a water flux of ~20 LMH in FO mode with a 1 M NaCl draw solution and DI water feed solution.

Table 6 lists the S-values of some commercial FO membranes tested by different researchers, and we have noted the transport methodology used to determine the permeability value. Differences in transport parameters for similar types of commercial membranes were noted due to the differences in operating and testing conditions. This further underscores the necessity of proposing a standard methodology and testing requirements to calculate the transport parameters of FO membranes.

## 5. Perspective and Conclusions

Unlike pressure-driven RO membranes, the support layer of FO membranes plays a critical role in reducing ICP effects and, therefore, providing high permeabilities. In this review, we have focused on the various fabrication techniques to create supports with low S-values, the methodologies used to quantify the intrinsic transport parameters, and finally, an overview of the commercial FO membranes and their performance. Among the three main techniques widely reported in the literature, electrospun FO supports had the lowest S-values. The solvent casting and hollow fiber spinning techniques, considered conventional for NF/RO processes, must be adapted specifically for FO.

This review notes the wide range of testing conditions and transport methodologies used to test FO membranes across different research labs, which makes comparison of these membranes extremely difficult. This point has been further emphasized by comparing the performance of a commercial membrane as reported by various labs and the reported data showed a significant level of variation in the S-values. Such variabilities in a “standard” material could affect the benchmarking of the performance of novel materials developed in labs. It is, therefore, critical to propose standard methodologies and testing requirements to calculate the transport parameters (A, B and S), and perhaps perform a multi-lab round-robin test similar to the one recently carried out by the gas separations community [145]. The latter will contribute to ensuring better reproducibility in results for FO membranes.

FO membranes are proposed for the treatment of hypersaline water produced where the temperature of the water is ≥90 °C [146], and it is our observation that very few research groups performed their membrane tests at these elevated temperatures. It is also deemed equally important to test FO membranes under hypersaline conditions instead of the 0.5–2 M NaCl solutions typically employed for most lab-scale tests. Although significant strides have been made in terms of membrane material development, we envision that more realistic performance analysis and the development of standardized methods of testing will contribute to the further growth of this field.

## Figures and Tables

**Table 1:** Operating conditions to determine the transport parameters (A, B, and *S*) of FO membrane (reproduced from Cath et al. [71] with permission from Elsevier).

(**a**)		
**Experimental Conditions**	**Value**	**Additional Notes**
Feed and DS temperature	20 °C	
DS concentration	1.0 M NaCl	58.44 g/L NaCl
Feed concentration	DI Water	
Feed and DS cross flow velocity	0.25 m/s	Feed and draw solutions’ flow rate defined by multiplying flow velocity with a cross sectional area of the flow channel perpendicular to flow direction
(**b**)		
**Experimental Conditions**	**Value**	**Additional Notes**
Feed temperature	20 °C	
Feed pressure	8.62 (125) bar (psi)	For high permeability membranes, 4.82 bar (70 psi) is recommended For both high and low permeability membranes, testing under more than one feed pressure is recommended to validate membrane integrity
Feed concentration	DI Water 2000 mg/L NaCl	Used deionized water to determine water permeability coefficient (*A*) Used NaCl solution for rejection test and determination of salt permeability coefficient (*B*)
Cross flow velocity	0.25 m/s	Similar to FO testing Preferably without any feed spacer

(**a**) **Testing mode:** Determination of *S* under FO mode; (**b**) **Testing mode:** RO for determination of *A* and *B*.

**Table 6:** Comparison in performance of commercial FO membranes as reported in different research articles. For better understanding, we designated the different methods used to calculate the intrinsic parameters (refer to the discussion in Section 2)—method #1 is pressurized RO-FO (Section 2.1.1a); method #2 is ultra-low-pressure RO-FO (Section 2.1.1b); and method #3 is single stage FO (Section 2.1.2).

Membrane	Water Permeability, A (LMH/bar)	*S*-Value (μm)	$\mathrm{Method}\;\mathrm{to}\;\mathrm{Calculate}\;A{\;}^{g}$	References
CTA-W ^a^	0.34 ^h^	950	#1	[141]
HTI-CTA ^a^	0.62	690	#3	[87]
HTI-CTA ^a^	0.44	481	#1	[142]
HTI-CTA ^a^	0.68	578	#1	[88]
HTI-CTA ^a^	0.36	595	#1	[100]
CTA-HW ^a^	1.19 ^h^	720	#1	[143]
HTI-CTA ^a^	0.55	463	#1	[144]
TFC-HTI ^b^	1.63	690	#1	[144]
TFC-Oasys ^d^	4.72	365	#1	[144]
HTI-M ^c^	0.64 ^h^	3074 ^i^	#1	[95]
TFC-Oasys ^d^	4.25	483	#1	[79]
Aquaporin FO ^e^	0.52	630	#1	[139]
Aquaporin ^f^	0.43	210	#3	[140]

^a^ Flat sheet cellulose triacetate-based FO membrane supplied by Hydration Technology Innovations. ^b^ Flat sheet TFC-based FO membrane supplied by Hydration Technology Innovations. ^c^ Flat sheet asymmetric cellulose triacetate-based FO membrane supplied by Hydration Technology Innovations. ^d^ Flat sheet FO membrane supplied by Oasys. ^e^ Flat sheet FO membrane supplied by Aquaporin A/S. ^f^ Hollow fiber FO membrane supplied by Aquaporin A/S. ^g^ The operating temperature for the RO and FO methods was 25 ± 5 °C. ^h^ Operating temperature for the RO was not recorded. ^i^ Operating temperature for the FO was not recorded.

## Nomenclature

FO mode/AL-FS	Active layer faces feed solution
Al_2_O_3_	Aluminum oxide nanoparticles
CNT	Carbon nanotubes
CA	Cellulose acetate
CAB	Cellulose acetate butyrate
P84 copolyimide	Copolyimide of 3,3′, 4,4′-benzophenone tetra-carboxylic dianhydride with 80% methylphenylene diamine and 20% methylene diamine
BPSH100-BPS0	Disulfonated poly(arylene ether sulfone) multiblock copolymer
ECP	External concentration polarization
FO	Forward osmosis
f-CNT	Functionalized multi-walled carbon nanotube
gMH	$g/m^{2}\;h\;$
GO	Graphene oxide
GP	Graphene oxide-graft-poly(2-hydroxy ethyl methacrylate) nanoparticles
HNT	Halloysite nanotubes
HTI	Hydration Technology Innovations
ICP	Internal concentration polarization
INTs	Imogolite nanotubes
LMH	$L/m^{2}\;h$
LMH/bar	$L/m^{2}\;h\;\mathrm{bar}$
MIP	Mercury intrusion porosimetry
Micro-XRM	Micro X-ray microscopy
MMcf	Million cubic feet
MPD	m-phenylene diamine
MWCNT	Multi-walled carbon nanotubes
NF	Nanofiltration
NMP	N-Methyl-2-pyrrolidone
DMF	N,N- dimethylformamide
OSRO	Osmotically assisted reverse osmosis
PAN	Polyacrylonitrile
PFSA	Perfluorosulfonic acid
PA-TFC	Polyamide thin-film composite
PEG	Polyethylene glycol
PET	Polyethylene terephthalate
PEI	Polyetherimide
PES	Polyethersulfone
PESU-co-sPPSU	Sulfonated copolymer of polyethersulfone and polyphenylsulfone
PDA	Polydopamine
PPSU	Poly(phenyl sulfone)
PSf	Polysulfone
PSf-g-PDMA	Polysulfone-graft-poly(2-dimethylaminoethyl methacrylate)
PVA	Polyvinyl alcohol
PVC	Polyvinyl chloride
PVDF	Polyvinylidene fluoride
PRO mode/AL-DS	Pressure retarded osmosis/Active layer faces draw solution
RO	Reverse osmosis
B	Salt permeability
SEM	Scanning electron microscopy
SPEK	Sulfonated poly(ether ketone)
SPES	Sulfonated polyethersulfone
SPPO	Sulfonated poly(phenylene oxide)
sPPSU	Sulfonated polyphenylenesulfone
SPSU	Sulfonated polysulfone
S-value	Structural parameter
TiO_2_	Titanium dioxide
TDS	Total dissolved solids
TFC	Thin-film composite
TMC	Trimesoyl chloride
A	Water permeability
NaY	Zeolite nanoparticles
Zn_2_GeO_4_	Zinc germanate
